# A Multi-Label Deep Learning Model for Detailed Classification of Alzheimer's Disease

**DOI:** 10.62641/aep.v53i1.1728

**Published:** 2025-01-05

**Authors:** Mei Yang, Yuanzhi Zhao, Haihang Yu, Shoulin Chen, Guosheng Gao, Da Li, Xiangping Wu, Ling Huang, Shuyuan Ye

**Affiliations:** ^1^Department of Psychiatry, Affiliated Kangning Hospital of Ningbo University, 315201 Ningbo, Zhejiang, China; ^2^Department of Psychiatry, Ningbo Kangning Hospital, 315201 Ningbo, Zhejiang, China; ^3^Department of Clinical Laboratory, Ningbo No.2 Hospital, 315099 Ningbo, Zhejiang, China; ^4^Department of Neurology, Ningbo No.2 Hospital, 315099 Ningbo, Zhejiang, China

**Keywords:** Alzheimer's disease, dot-product attention mechanism, diagnostic accuracy, disease subtypes, precision medicine, artificial intelligence

## Abstract

**Background:**

Accurate diagnosis and classification of Alzheimer's disease (AD) are crucial for effective treatment and management. Traditional diagnostic models, largely based on binary classification systems, fail to adequately capture the complexities and variations across different stages and subtypes of AD, limiting their clinical utility.

**Methods:**

We developed a deep learning model integrating a dot-product attention mechanism and an innovative labeling system to enhance the diagnosis and classification of AD subtypes and severity levels. This model processed various clinical and demographic data, emphasizing the most relevant features for AD diagnosis. The approach emphasized precision in identifying disease subtypes and predicting their severity through advanced computational techniques that mimic expert clinical decision-making.

**Results:**

Comparative tests against a baseline fully connected neural network demonstrated that our proposed model significantly improved diagnostic accuracy. Our model achieved an accuracy of 83.1% for identifying AD subtypes, compared to 72.9% by the baseline. In severity prediction, our model reached an accuracy of 83.3%, outperforming the baseline (73.5%).

**Conclusions:**

The incorporation of a dot-product attention mechanism and a tailored labeling system in our model significantly enhances the accuracy of diagnosing and classifying AD. This improvement highlights the potential of the model to support personalized treatment strategies and advance precision medicine in neurodegenerative diseases.

## Introduction

Alzheimer’s disease (AD) is a progressive neurodegenerative disorder that presents significant challenges to global healthcare. It is the leading cause of dementia in older adults, characterized by the gradual deterioration of cognitive functions, including memory, thinking, and behavior. The complexity of AD arises from its diverse manifestations, ranging from mild cognitive impairment to severe dementia, making its diagnosis and management particularly challenging [1].

The pathophysiology of AD involves the accumulation of amyloid-beta plaques and tau tangles in the brain, leading to neuronal damage and loss. Clinically, the disease presents with symptoms such as memory loss, confusion, impaired judgment, personality changes, and difficulties in performing daily activities, which worsen over time, severely impacting the quality of life of patients and their families [2].

AD subtypes include the Logopenic Variant, characterized by difficulties in word retrieval and sentence repetition; Posterior Cortical Atrophy, marked by visual processing deficits and other posterior brain functions; and Frontal Variant, involving behavioral changes and executive dysfunction. Treatment for these subtypes varies: the Logopenic Variant often requires speech and language therapy, Posterior Cortical Atrophy involves management with visual aids and occupational therapy, and the Frontal Variant focuses on behavioral interventions and medications targeting psychiatric symptoms alongside supportive therapies like cognitive stimulation and physical activity [3]. Accurate diagnosis and classification of AD are crucial for effective treatment and management, as highlighted by Beach *et al*. [4].

Traditional diagnostic methods, which rely on cognitive tests and imaging, are limited in capturing the nuanced progression and subtypes of AD. This often results in a generalized treatment approach that fails to effectively address the individual needs of a patient [5]. Recently, non-imaging biomarkers like genetics, cerebrospinal fluid (CSF), and blood-based markers, have emerged as valuable tools for enhancing diagnostic accuracy [6]. These non-invasive diagnostic tools and attention mechanism advances offer significant promise for improving the accuracy and efficiency of disease diagnosis, particularly in medical imaging [7].

This study aimed to develop a model that integrates a dot-product attention mechanism with an innovative labeling system to enhance the precision of AD diagnosis. By selectively focusing on the most clinically relevant data, the model replicates the decision-making processes of expert clinicians, leading to improved identification of AD subtypes and their severity. This refined diagnostic capability enables the creation of personalized treatment plans tailored to patients’ specific needs, advancing precision medicine in the management of neurodegenerative diseases [8].

### Related Work

In the past decade, the application of deep learning in medical diagnostics has seen significant growth, especially in AD. Numerous machine learning and deep learning techniques have been investigated for their potential in the early diagnosis and classification of AD, reflecting a growing interest in leveraging technology to enhance clinical practices.

#### Binary Classification Models

Early studies, such as those by Suk *et al*. [9] and Lu *et al*. [10], utilized Magnetic Resonance Imaging (MRI) and Positron Emission Tomography (PET) in deep learning models to distinguish AD patients from healthy controls, achieving significant diagnostic accuracy. However, these binary classification models mostly oversimplify the complexity of AD by focusing solely on the presence or absence of the disease without accounting for its diverse manifestations. Our study advances this approach by incorporating a dot-product attention mechanism, which enhances the ability of the model to focus on the most relevant features for AD diagnosis, thereby improving classification performance.

#### Multi-Class Classification Models

To address the limitations of binary models, multi-class classification approaches have been developed to capture the progression of AD effectively. For example, Ding *et al*. [11] employed Convolutional Neural Networks (CNNs) to classify individuals into categories such as cognitively normal, mild cognitive impairment (MCI), and AD, using multi-modal neuroimaging data. While these models provide deeper insights into disease stages, their reliance on extensive imaging data limits their practicality in diverse clinical settings. Our approach builds on this by integrating advanced attention mechanisms, improving the interpretability and accuracy of multi-class classification models.

#### Incorporation of Non-Imaging Biomarkers

Recent studies have expanded diagnostic approaches to include non-imaging biomarkers such as genetic data, cerebrospinal fluid (CSF) analysis, and blood-based markers, as explored by Ou *et al*. [12] and Klyucherev *et al*. [13]. These studies highlight the potential of these non-invasive tools that predict AD, offering significant enhancements in diagnostic capabilities. Our study incorporates these advancements by adopting a multi-modal approach that combines imaging and non-imaging biomarkers, enhancing the robustness and accuracy of AD diagnostics.

#### Multi-Modal Deep Learning Approaches

Further advancements have been made with multi-modal deep learning frameworks, such as those proposed by Lin *et al*. [14], which integrate imaging, genetic, and clinical data. These comprehensive approaches, incorporating cognitive assessments and lifestyle factors, provide a holistic view of the patient, thus improving diagnostic accuracy [15, 16, 17]. Our study builds on these methodologies by integrating a dot-product attention mechanism, further enhancing the interpretability and accuracy of multi-modal AD diagnosis models.

#### Advances in Data Integration and Model Interpretability

The integration of diverse data sources and the enhancement of model interpretability through techniques like attention mechanisms and explainable AI (XAI) have been crucial in improving the transparency and credibility of artificial intelligence (AI) models in clinical settings [18, 19]. These models help clinicians understand and trust the decision-making processes of AI systems. Our model incorporates these techniques, ensuring that its decision-making process is transparent and consistent with clinical expertise.

#### Emerging Technologies and Future Directions

Emerging technologies such as federated learning and transfer learning address challenges related to data availability and privacy, promoting more collaborative and adaptable research environment [20, 21, 22, 23]. These technologies hold the potential to make AI-driven diagnostics more accurate, accessible, and secure, paving the way for future advancements in AD diagnosis.

#### Challenges and Limitations

Despite these advancements, challenges remain, including the heterogeneity of data sources and the difficulty in acquiring large, well-annotated datasets. Additionally, many studies focus on broad AD categorizations, which may not fully capture the nuanced classification of disease stages and subtypes.

### Our Contribution

Our research advances the field by developing a multi-label deep learning model capable of distinguishing between AD and non-AD subjects and classifying detailed AD stages and subtypes. By leveraging a comprehensive dataset encompassing demographic, hematological, biochemical, endocrine, immunological, and neurological markers, we aimed to elucidate the complex interrelationships among these factors. Our modular neural network architecture processes these data categories independently before integrating them to enhance diagnostic accuracy and generalization. This model represents a significant step toward precision medicine in neurodegenerative diseases, offering robust tools for early detection and personalized treatment strategies.

## Materials and Methods

### Overview

**Fig. 1.**
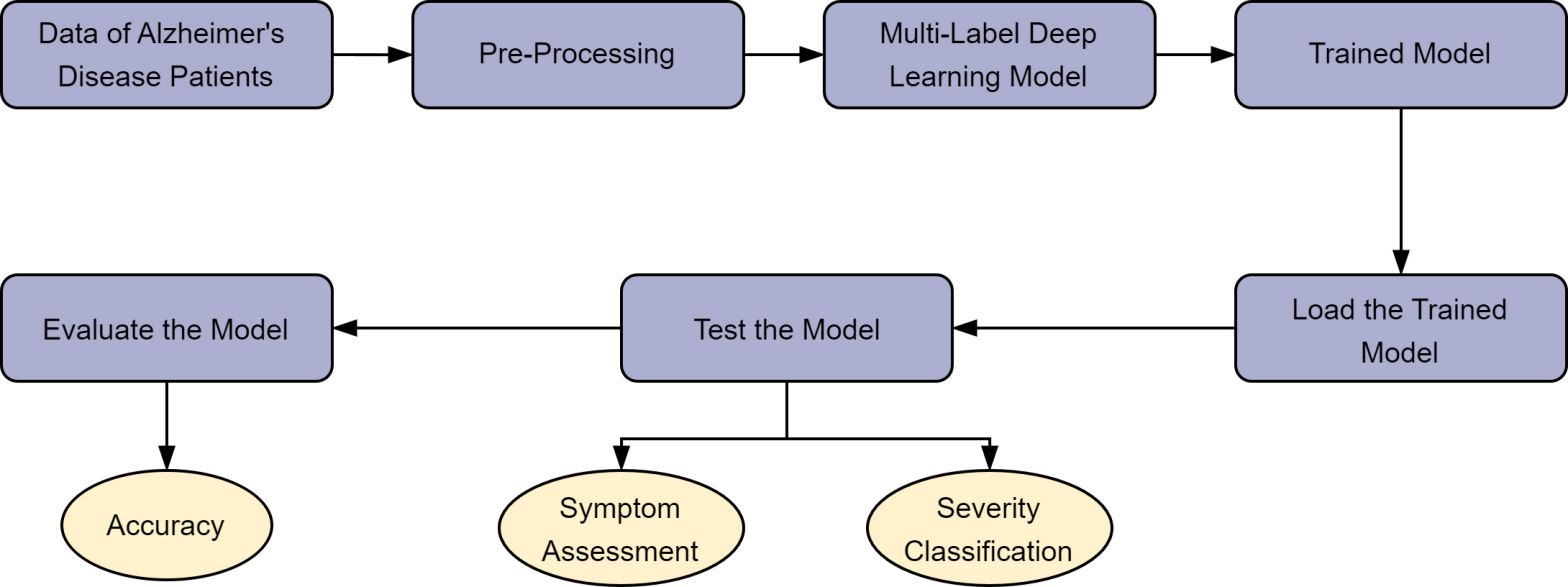
**Research flowchart (created with Lark (version 7.21.6, Beijing Bytedance technology company Limited, Beijing, China))**. A summary of the research process for developing a modular neural network with dot-product attention mechanisms. The flowchart outlines steps from data collection and preprocessing to feature extraction via attention mechanisms, culminating in the integration and prediction of Alzheimer’s disease (AD) subtypes and severity.

We developed a modular neural network architecture that utilizes dot-product attention mechanisms to analyze and predict various AD types based on clinical and demographic data. Our methodology involves categorizing the data into specific groups, applying attention mechanisms to extract key features from each group, and integrating these features to form a comprehensive prediction. The research process is systematically illustrated in Fig. 1, which outlines the steps from data collection and pre-processing to applying attention mechanisms and the final prediction stages.

### Data Categorization

To facilitate detailed analysis, we organized the data into the following categories based on their relevance to diagnosing and understanding Alzheimer’s disease (AD):

∙ Sex and Age: Basic demographic variables critical for analyzing AD distribution and progression.

∙ Blood Chemistry and Hematology: Including white blood cell count and subtypes (White Blood Cell (WBC), Neutrophils (NEU), Lymphocytes (LYM), Monocytes (MONO), Eosinophils (ESO), Basophils (BASO)), red blood cell count and related parameters Red Blood Cell (RBC), Hemoglobin (HGB), Hematocrit (HCT), Mean Corpuscular Volume (MCV), Mean Corpuscular Hemoglobin (MCH), Mean Corpuscular Hemoglobin Concentration (MCHC), and platelet count and related parameters (Platelets (PLT), Mean Platelet Volume (MPV), Platelet Distribution Width (PDW), Procalcitonin (PCT)). These parameters provide insights into general health and can be associated with neurodegenerative processes.

∙ Biochemical Markers: Encompassing liver function indicators (Total Bilirubin (TBIL), Direct Bilirubin (DBIL), Indirect Bilirubin (IDBIL), Total Protein (TP), Albumin (ALB), Globulin (GLB), Albumin to Globulin Ratio (AG_ratio)), liver and heart enzymes (Alanine Aminotransferase (ALT), Aspartate Aminotransferase (AST), Lactate Dehydrogenase (LDH), Gamma-Glutamyl Transferase (GGT), Alkaline Phosphatase (ALP)), blood glucose and lipids (Glucose (GLU), Triglycerides (TG), Total Cholesterol (TCHO), High-Density Lipoprotein Cholesterol (HDLC), Low-Density Lipoprotein Cholesterol (LDLC), Apolipoprotein A-1 (APOA1), Apolipoprotein B (APOB), Apolipoprotein E (APOE)), electrolytes and minerals (Potassium (K), Sodium (Na), Chloride (Cl), Calcium (Ca), Phosphorus (P), Magnesium (Mg), Iron (Fe)), kidney function markers (Urea (UREA), Creatinine (CR), Uric Acid (UA)), inflammation markers (Homocysteine (HCY), C-Reactive Protein (CRP)), and vitamins (Vitamin B12 (VB12), Folic Acid (Folicacid)), all of which are relevant to cognitive functions.

∙ Endocrine and Immunological Markers: Including immunoglobulins and complement system components (Immunoglobulin A (IGA), Immunoglobulin G (IGG), Immunoglobulin M (IGM), Complement Component 3 (C3), Complement Component 4 (C4)), thyroid function tests (Thyroid-Stimulating Hormone (TSH), Triiodothyronine (T3), Thyroxine (T4), Free Triiodothyronine (FT3), Free Thyroxine (FT4), Thyroid Peroxidase Antibodies (TPOAb), Thyroglobulin Antibodies (TGAb)), and other hormones and cancer markers (Prolactin (PRL), Alpha-Fetoprotein (AFP), Carcinoembryonic Antigen (CEA), Ferritin (FER), Carbohydrate Antigen 19-9 (CA19-9)).

∙ Neurological Markers: β2-Microglobulin (β2mg), a potential neurodegenerative disease indicator, and the Mini-Mental State Examination (MMSE) [24], essential for assessing cognitive function.

∙ Lifestyle and Demographic Factors: Marital status, education level, smoking, alcohol consumption, diabetes, hypertension, coronary heart disease, and activities of daily living (Marriage, education, smoking, alcohol, Diabetes Mellitus (DM), Hypertension (HT), Coronary Heart Disease (CHD), and Activities of Daily Living (ADL)), which provide insights into the lifestyle and socioeconomic factors of the patient affecting disease outcomes.

### Data Pre-processing

We addressed missing values and sample imbalance during pre-processing to ensure data quality and optimize model performance. We applied mean imputation to handle missing values since they were minimal and randomly distributed. Specifically, missing values for each feature were replaced with the mean of that feature, preserving dataset consistency and minimizing the impact on model training.

To address sample imbalance, we used the Synthetic Minority Over-sampling Technique (SMOTE), which generates new minority class samples, improving the performance of the model on underrepresented classes. SMOTE was applied specifically to the training set to enhance the learning and generalization of the model.

All relevant AD factors were normalized to ensure consistency within the model. Each factor was scaled to a 0–1 range. For example, for β2mg (with a typical range of 1–3 mg/L), 1 mg/L was normalized to 0 and 3mg/L to 1. This normalization facilitates consistent comparison and computation of different factors within the model.

### Inclusion and Exclusion Criteria

#### Inclusion Criteria

∙ Patients aged 50 years and older.

∙ Diagnosed with AD or presenting symptoms suggestive of dementia.

∙ Comprehensive clinical records are available, including complete blood work and medical history.

#### Exclusion Criteria

∙ Patients with other types of dementia, such as vascular dementia or dementia with Lewy bodies.

∙ Incomplete datasets or missing critical health information.

∙ Recent history of substance abuse or conditions mimicking dementia symptoms, such as severe vitamin deficiencies or thyroid dysfunction.

### Integration and Processing

Data were consolidated into four main groups for input into our model: Blood Chemistry, Biochemical Markers, Endocrine and Immunological Markers, and Lifestyle and Demographic Factors. This structure allows for targeted processing, enhancing the capacity of the model to identify significant predictive features. Critical indicators such as β2mg and MMSE were directly fed into the model due to their direct relevance to AD diagnosis [25].

The model utilizes a dot-product attention mechanism to focus on significant data features, calculating attention scores to emphasize the most informative aspects for AD prediction. This technique enhances the accuracy and relevance of the model in clinical settings.

### Target Definition

The final target variable was the predicted stage and subtype of AD, derived from the integrated analysis of the categorized data. This approach ensures that the model outputs are clinically applicable, supporting personalized treatment and management plans for AD patients.

### Dot-Product Attention Mechanism

**Fig. 2.**
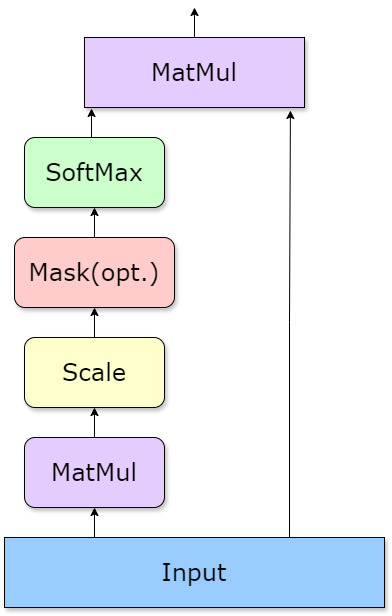
**Dot-product attention workflow (created with Lark (version 7.21.6, Beijing Bytedance technology company Limited, Beijing, China))**. The figure illustrates the workflow of the dot-product attention mechanism, which enhances model precision by focusing on critical clinical data features. This process is key to accurately identifying different AD subtypes and stages. MatMul, Matrix Multiplication.

Our model employs a dot-product attention mechanism, as illustrated in Fig. 2, to enhance AD analysis. This technique allows the model to focus on the most relevant clinical data, improving its capacity to accurately identify AD subtypes and stages.

#### Process Overview

(1) Input Transformation: Clinical data, such as Blood Chemistry and Biochemical Markers, were transformed into a 1 × n vector format. This simplified representation facilitates processing within the attention mechanism.

(2) Attention Scores: The model calculates attention scores by performing a dot product operation on the input vector with itself. These scores were then scaled to maintain their interpretability and significance.

(3) Context Vector: Using the attention scores, the model generates a context vector, a summary that captures the most critical information from the input data. This is achieved by computing a weighted sum of the data features, with weights derived from the normalized attention scores.

#### Mathematical Description

The dot-product attention mechanism is mathematically represented as follows:

$$\mathrm{Attention}\left(X\right)=\mathrm{softmax}\left(\frac{X\,X^{T}}{d_{k}}\right)\,X$$

Here, X represents the input vector, and d_k_ is a scaling factor that stabilizes the magnitude of the attention scores. This mechanism enables the model to effectively process diverse clinical data, leading to more accurate and detailed AD diagnoses, including subtypes and severity assessments.

### Network Architecture

The proposed network architecture consists of multiple specialized modules, each tailored to handle specific data types, such as Blood Chemistry and Biochemical Markers. Each module employs the dot-product attention mechanism to process and highlight the most relevant information, resulting in a context vector for each data category.

#### Steps of the Architecture

**Fig. 3.**
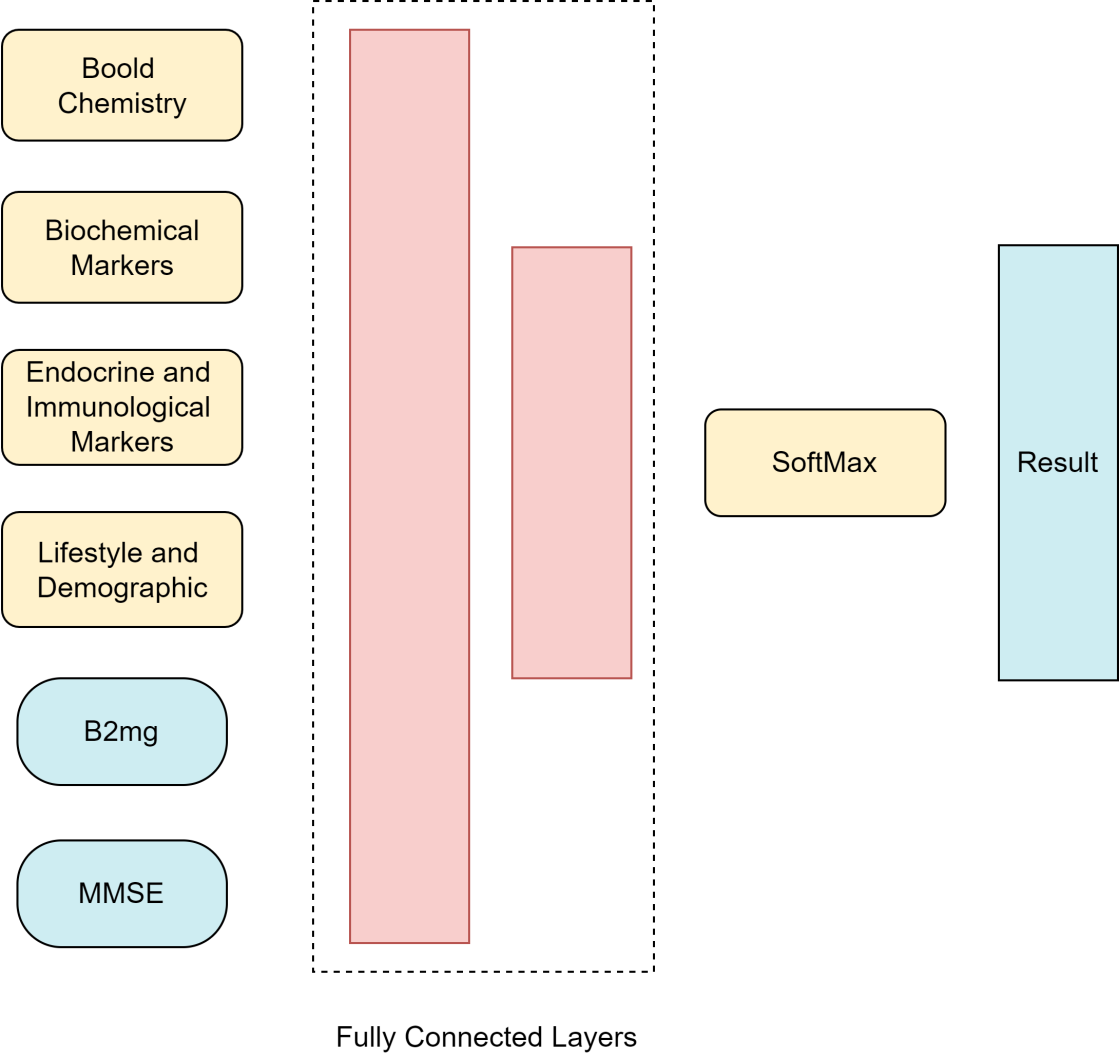
**Structure of the proposed method (created with Lark (version 7.21.6, Beijing Bytedance technology company Limited, Beijing, China))**. It outlines the structure of the proposed method, detailing steps from input processing and attention mechanism application to context vector integration and final prediction using fully connected layers. This structure improves the accuracy of AD subtype and severity predictions. B2mg, β2-Microglobulin; MMSE, Mini-Mental State Examination.

The architecture operates through the following steps (Fig. 3):

(1) Input Processing: Data from each category is input into its respective module. Complex datasets with multiple indicators are decomposed, while simpler datasets with single indicators, like β2mg or MMSE, are processed directly.

(2) Attention Mechanism: The attention mechanism extracts key features from each module by computing attention scores. These scores are then normalized to ensure consistency and meaningful results.

(3) Context Vector Combination: The context vectors from all modules are combined into a single comprehensive feature vector, ensuring a uniform representation of all data categories.

(4) Fully Connected Layers: The combined feature vector passes through several fully connected layers, culminating in a softmax layer that predicts the type and severity of AD.

This architecture enables the model to focus on the most significant data features, improving prediction accuracy.

### Label Design

The model incorporates a novel labeling system that assesses AD severity and differentiates between its subtypes. It outputs three values, each ranging from 0 and 1, corresponding to the following AD subtypes [26]:

(1) Logopenic Variant: Primarily associated with language impairment.

(2) Posterior Cortical Atrophy: Affecting visual processing.

(3) Frontal Variant: Involving changes in behavior and personality.

The severity of each subtype is calculated, and overall disease severity is determined by averaging these values. This labeling system allows for detailed and nuanced predictions, enhancing the diagnostic accuracy of the model and providing valuable insights into AD progression.

## Experiments and Evaluation

In this section, we outline the experimental setup and evaluation methods to demonstrate the effectiveness of our proposed model compared to a baseline model. We aimed to highlight the improvements gained from integrating a dot-product attention mechanism and an innovative labeling system for detailed analysis and classification of AD.

### Experimental Setup

We utilized a comprehensive dataset comprising clinical and demographic data, including sex, age, blood chemistry, hematology, biochemical markers, endocrine and immunological markers, neurological markers, and lifestyle factors. Data were collected from 430 eligible patients at the Affiliated Kangning Hospital of Ningbo University between January 2022 and December 2023. The dataset was divided as follows: 70% for training, 15% for validation, and 15% for testing.

Two models were compared in our experiments:

(1) Baseline model: A fully connected neural network with a similar number of layers and parameters as our proposed model but without specialized data processing mechanisms.

(2) Proposed model: Incorporating a dot-product attention mechanism to enhance data processing. This model specifically outputs three values estimating the severity of different AD subtypes. These values were averaged to compute an overall severity score.

### Evaluation Metrics

We employed accuracy as our primary metric. This involved:

∙ Checking the accuracy with which each model identified AD subtypes and severity levels.

∙ Evaluating subtype accuracy by determining if the model correctly identified each subtype, such as the Logopenic Variant, Posterior Cortical Atrophy, and Frontal Variant, with an output value threshold of 0.5 indicating the presence of the subtype.

∙ Measuring severity accuracy by defining specific thresholds for different severity levels of the disease—0.25 for asymptomatic, 0.5 for mild, 0.75 for moderate, and 1.0 for severe—and comparing the model’s predictions against these benchmarks.

### Statistical Analysis

We utilized GraphPad 8.0 software (GraphPad Software LLC, San Diego, CA, USA) for statistical analysis. Descriptive statistics, including mean, standard deviation, minimum, and maximum values, were used to summarize the data. Differences between groups were analyzed using the chi-square test for categorical data and the *t*-test for quantitative data. Data were presented as mean ± standard deviation for normally distributed data and median (P25, P75) for non-normally distributed data.

## Results

### Patient Demographic Data

In our study, we compared the performance of our proposed model, which incorporates a dot-product attention mechanism, with a traditional baseline neural network. The evaluation focused on the accuracy of identifying AD subtypes and the precision in predicting disease severity. To ensure a thorough analysis, we included a detailed demographic and clinical profile of the patients, encompassing variables such as age, sex, marital status, body mass index (BMI), disease duration, smoking and drinking history, and medical histories of diabetes and hypertension. These characteristics are summarized in Table 1 and were included to control for potential confounders, enhancing the robustness of the performance assessment for our model.

**Table 1.** **Patient demographics**.

Characteristic		Value (n = 430)
Age–year		77.22 ± 8.87
Female sex–no. (%)		215 (50.00)
Marital status–married. (%)		254 (59.07)
Body mass index		24.83 ± 7.16
Duration of disease–year		5.5 (7.6, 16.4)^a^
Smoking and drinking history–no. (%)		
	Smoking history–no. (%)	91 (21.16)
	Drinking history–no. (%)	48 (11.16)
History of diabetes–no. (%)		245 (59.98)
History of hypertension–no. (%)		49 (11.40)

Note: (a) Median (P25, P75).

### Comparison of Training, Validation, and Testing Results

The performance of the model was evaluated across the training, validation, and testing datasets using key metrics: accuracy, precision, recall, and F1 score (Table 2). The results indicate consistent performance across all datasets, with only slight variations in these metrics. This consistency suggests that the model generalizes well and is robust in predicting AD subtypes and severity across different datasets.

**Table 2.** **Comparison of model performance across training, validation, and testing sets**.

Dataset	Accuracy	Precision	Recall	F1 score
Training	92%	90%	91%	90.5%
Validation	90%	88%	89%	88.5%
Testing	89%	87%	88%	87.5%

### Model Performance in Recognizing AD Subtypes

We evaluated the ability of the model to recognize different AD subtypes using accuracy, precision, recall, and F1 score, with results summarized in Table 3. Additionally, confusion matrices for each subtype provide a visual representation of the performance of the model, showing the frequency of correct and incorrect classifications. These matrices help identify the strengths and areas for improvement in the model.

**Table 3.** **Model performance in AD subtype classification**.

AD subtype	Accuracy	Precision	Recall	F1 score
Logopenic Variant	89%	87%	85%	86%
Posterior Cortical Atrophy	91%	90%	88%	89%
Frontal Variant	88%	86%	84%	85%

### Subtype Accuracy

The accuracy of correctly identifying AD subtypes in the test set is summarized in Table 4. The proposed model demonstrated significant improvements in recognizing subtypes compared to the baseline model. Specifically, accuracy increased to 81.4% for the Logopenic Variant, 83.6% for Posterior Cortical Atrophy, and 84.2% for the Frontal Variant, compared to 68.4%, 74.0%, and 76.3%, respectively, with the baseline model. Overall accuracy improved from 72.9% with the baseline model to 83.1% with the proposed model. These enhancements are crucial for clinical practice as they enable more precise diagnoses of the specific AD subtype, essential for determining the most effective treatment approach and management plan.

**Table 4.** **Accuracy comparison for AD subtype identification between the baseline and the proposed models**.

Model	Logopenic Variant	Posterior Cortical Atrophy	Frontal Variant	Overall accuracy
Baseline model	68.4%	74.0%	76.3%	72.9%
Proposed model	81.4%	83.6%	84.2%	83.1%

### Severity Accuracy 

The accuracy of predicting AD severity levels is summarized in Table 5. The proposed model outperformed the baseline model, accurately predicting the asymptomatic stage at 86.4%, mild severity at 83.6%, moderate severity at 82.2%, and severe stages at 81.1%. These figures represent significant improvements over the accuracies from the baseline model at 78.2%, 74.3%, 71.5%, and 70.1%, respectively. The overall accuracy for severity prediction increased from 73.5% to 83.3%. Accurate severity assessment is crucial for tailoring treatment plans, impacting patient care and prognosis.

**Table 5.** **Accuracy comparison for AD severity prediction between the baseline and the proposed models**.

Model	Asymptomatic	Mild	Moderate	Severe	Overall accuracy
Baseline model	78.2%	74.3%	71.5%	70.1%	73.5%
Proposed model	86.4%	83.6%	82.2%	81.1%	83.3%

The results suggest that the proposed model significantly enhances diagnostic accuracy for AD in terms of subtype identification and severity prediction. This improvement has substantial implications for clinical practice, providing clinicians with more accurate diagnostic tools that facilitate personalized treatment strategies, ensuring that interventions are appropriately matched to the specific condition of the patient and disease stage.

## Discussion

Our study reveals that the proposed model, incorporating a dot-product attention mechanism, significantly outperforms the traditional baseline neural network in diagnostic accuracy. This improvement is evident in the superior ability of the model to identify AD subtypes and accurately predict disease severity.

The enhanced performance of our model can be primarily attributed to the structural benefits of the attention mechanism. Unlike conventional models that uniformly process data, our model selectively emphasizes the most informative features. This selective focus is crucial because AD manifests differently in patients, affecting them in diverse ways that standard models may not capture effectively. Previous research has indicated that attention mechanisms can significantly enhance the interpretability and performance of neural networks in medical diagnosis tasks [27, 28].

In practical terms, the attention mechanism functions similarly to a skilled clinician who, through experience, prioritizes specific symptoms or patient history details over others. By simulating this selective focus, the model processes data and interprets it in a clinically relevant manner. This approach leads to more precise predictions of the type severity of AD, which is invaluable in clinical settings. Such precision aids healthcare providers in developing personalized treatment plans tailored to the unique needs of each patient.

Moreover, accurately classifying the disease subtype and predicting its progression enable earlier and more targeted interventions, which are crucial for effective AD management. Early and precise interventions can significantly alter the disease trajectory, improving patient outcomes and quality of life, as supported by recent studies [29, 30].

Our proposed model advances the goal of precision medicine in AD care, where treatments are customized to the nuances of conditions for each patient. This approach enhances treatment efficacy and optimizes resource allocation within healthcare systems, ensuring appropriate treatments are delivered to patients at the right time. Emerging evidence supports the potential of such personalized medicine in revolutionizing chronic disease management [31].

However, several limitations of our model must be acknowledged. First, the performance of the model heavily depends on the quality and diversity of the input data. Biases or inconsistencies in the clinical and demographic data used for training could impact the generalizability of the model.

Second, despite integrating advanced computational techniques and the dot-product attention mechanism, the interpretability of the model remains challenging. The complexity of deep learning models can limit the ability of clinicians to fully trust and adopt these systems in practice.

Third, our study primarily utilizes retrospective data, and prospective validation in diverse, real-world clinical settings is necessary to confirm the efficacy and robustness of the model. Additionally, the model was trained and tested on datasets predominantly consisting of patients diagnosed with specific AD subtypes. Extending this approach to a more heterogeneous population with various neurodegenerative conditions could reveal further insights and potential limitations.

Finally, while our model shows promise in enhancing diagnostic precision, it does not yet incorporate longitudinal data to track disease progression over time. Future work should focus on integrating longitudinal datasets to improve the ability of the model to predict disease trajectory and treatment outcomes.

## Conclusions

Our study introduces a model enhanced by an attention mechanism and a sophisticated labeling system, significantly improving the diagnosis and classification of AD subtypes and severity levels. Experimental results demonstrate that this model outperforms traditional neural networks in accuracy. This advancement underscores the potential of our approach to providing more precise and clinically relevant diagnoses, supporting the development of targeted treatments and management strategies for AD.

## Availability of Data and Materials

The datasets used during the current study are available from the corresponding author on reasonable request.
